# Case report: EBV-positive epithelioid follicular dendritic cell sarcoma with CD30 expression: a highly challenging diagnosis

**DOI:** 10.3389/fonc.2023.1321565

**Published:** 2024-01-03

**Authors:** Ying Chen, Xin He, Xingyan Zhu, Yujuan Xu, Deyu Guo

**Affiliations:** Departments of Pathology, Guiqian International General Hospital, Guiyang, Guizhou, China

**Keywords:** follicular dendritic cell sarcoma, epithelioid, Epstein-Barr virus, CD30, differential diagnosis

## Abstract

**Introduction:**

Follicular dendritic cell sarcoma (FDCS) is a rare tumor entity with a wide range of anatomical sites and strong heterogeneity in morphology and immunohistochemistry, making it highly susceptible to misdiagnosis. There are two types of FDCS: conventional FDCS and EBV+ inflammatory FDCS. It is currently suggested that the former has nothing to do with EBV infection. Moreover, they have distinctively different clinicopathological characteristics.

**Case description:**

A 69-year-old male patient was admitted to our hospital after 4 months of progressive enlargement of the neck mass. Positron emission tomography/computed tomography (PET/CT) examination showed multiple enlarged lymph nodes in the body. After cervical lymph node excision and biopsy, it was found that the tumor cells were epithelioid and diffusely expressed EBER and CD30. It was initially diagnosed as poorly differentiated cancer and lymphoma. In subsequent differential diagnosis, we found that it strongly stained CD21 and CD23, which was approved the diagnosis of EBV+ FDCS.

**Conclusion:**

Epithelioid FDCS is very rare. EBV-positive FDCS with abnormal expression of CD30 has not been reported. Whether EBV also plays an important role in conventional FDCS requires more cases to be verified. Our case provides valuable research clues for further understanding the pathological characteristics of this tumor entity.

## Introduction

Follicular dendritic cell sarcoma (FDCS) is a rare tumor with morphological and immunophenotypic characteristics of FDC differentiation. It can occur in lymph nodes and a wide range of extranodal anatomical sites (1). The neoplastic cells have a variety of shapes, ranging from spindle or oval to epithelioid, anaplastic, and multinucleated. Due to a wide range of anatomical sites and strong heterogeneity in morphology and immunohistochemistry, it is highly susceptible to misdiagnosis. There are two types of FDCS, namely, conventional FDCS and EBV+ inflammatory FDCS, in the category of stroma-derived neoplasms of lymphoid tissues in the fifth edition of the World Health Organization Classification of Haematolymphoid Tumors (2). At present, it is generally accepted that conventional FDCS are negative for EBV, whereas the EBV+ inflammatory FDCS variant consistently shows EBV in the neoplastic cells (3). They have distinctive different clinicopathological characteristics. Herein, we report a case of EBV+ conventional FDCS with unusual morphology and diffusely expressed CD30. To the best of our knowledge, this phenomenon has never been reported before. This information will undoubtedly enhance our comprehension of FDCS molecular pathogenesis.

## Case description

A 69-year-old male patient was admitted to our hospital after 4 months of progressive enlargement of the neck mass. Two months ago, a puncture biopsy was performed in an external hospital and diagnosed as plasmablastic lymphoma (PBL). After consultation with another hospital, it was considered as metastatic poorly differentiated cancer of lymph node, and investigating the nasopharynx was recommended. Positron emission tomography/computed tomography (PET/CT) examination of the external hospital showed multiple enlarged lymph nodes in the left parapharyngeal space, left submandibular area, left neck, left supraclavicular fossa, right diaphragmatic angle area, hepatogastric space, hilar area, and retroperitoneal area (specific details were not available).On examination, multiple fused enlarged lymph nodes can be palpated in the left neck, with an ill-defined margin. Computed tomography (CT) revealed multiple enlarged lymph nodes in bilateral neck, supraclavicular fossa, posterior cervical space, and left parapharyngeal space, partially fused, with the largest being located in the left neck, approximately 35 mm × 31 mm. After completion of relevant preoperative examinations, left neck mass resection was performed. Following surgical resection 2 weeks later, he received two cycles of chemotherapy with cyclophophamide, doxorubicin, vincristine, and prednisolone (CHOP). Although the enlarged lymph nodes throughout the patient’s body still existed, they did not continue to enlarge. However, the patient refused subsequent chemotherapy and was discharged due to bone marrow suppression after chemotherapy.

### Pathological changes

**Figure 1:**
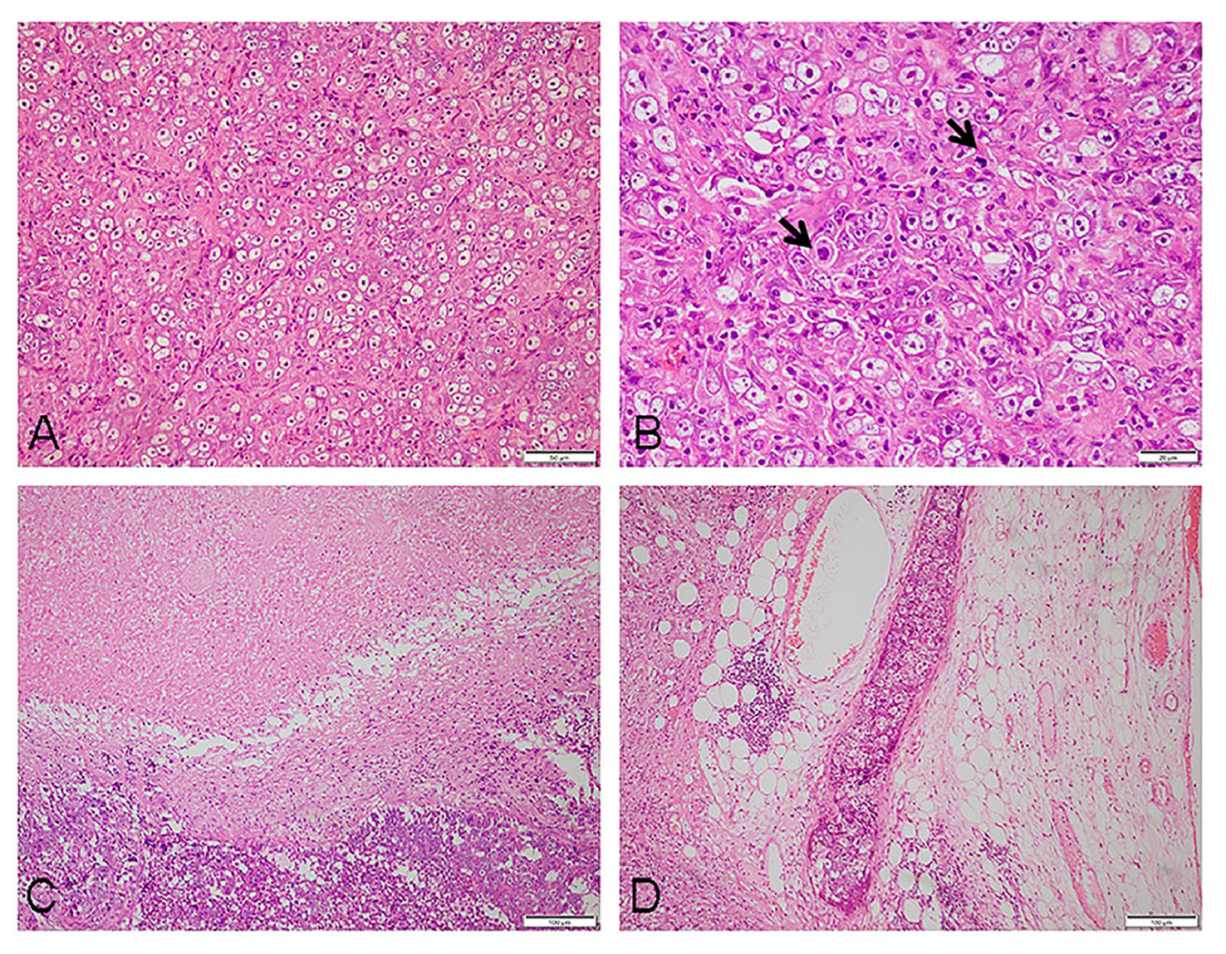
Histomorphology of the tumor: **(A)** at low magnification, the tumor cells appeared to be epithelioid, arranged in patches and nests (×200). **(B)** The tumor cells were large in size, the cytoplasm brightly eosinophilic, nuclei prominent, oval with coarse chromatin, and distinct nucleoli; mitotic figures were frequently seen (as shown by the arrow) (×400). **(C)** Visible tumor necrosis (×100). **(D)** Intravascular tumor thrombi can be found in the adipose tissue surrounding the tumor (×100).

**Figure 2:**
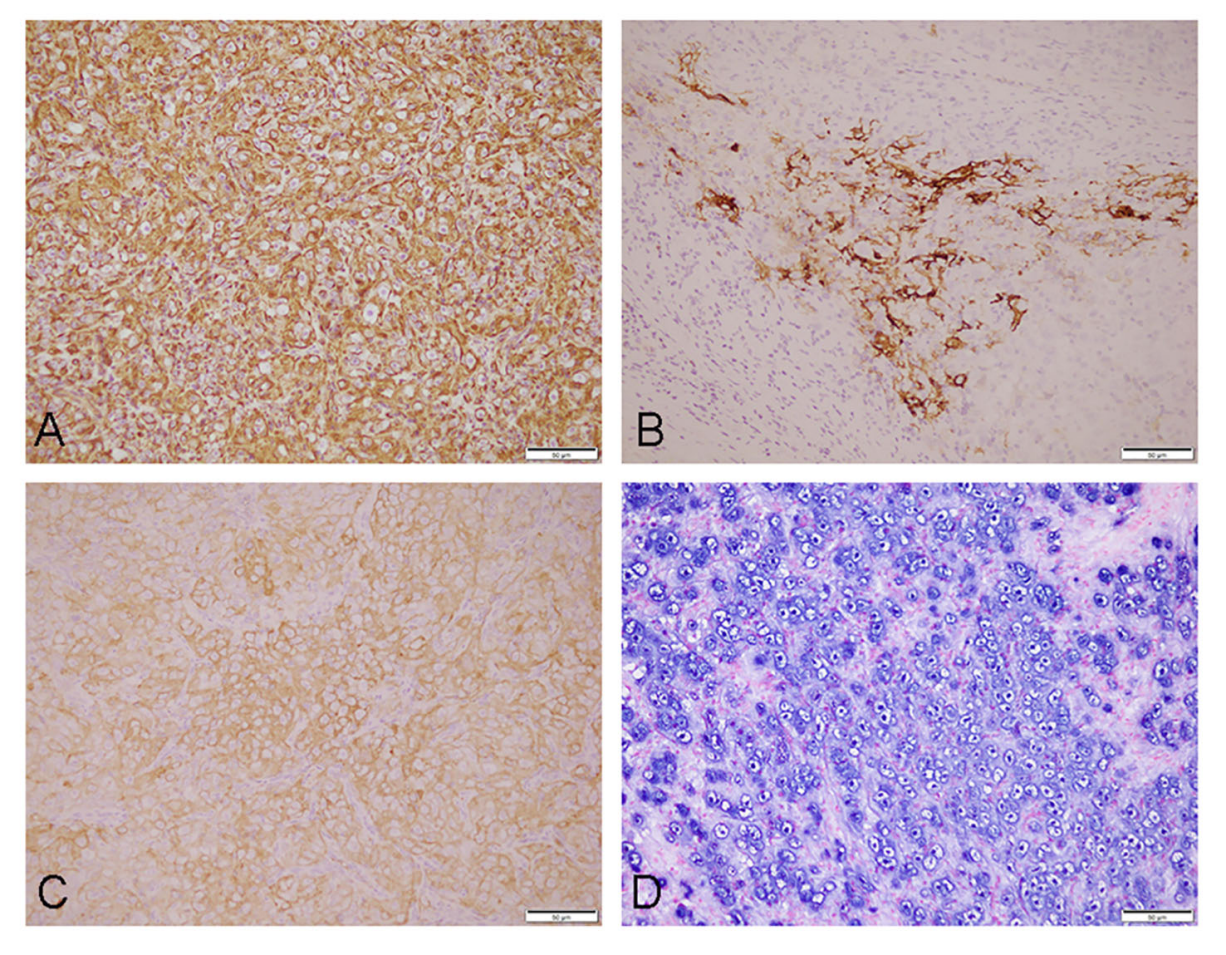
Immunophenotype and *in situ* hybridization features of the tumor: tumor cells immunohistochemistry staining results [**(A)**, CD21; **(B)**, CD23; and **(C)**, CD30]; *in situ* hybridization result of EBER **(D)**, all magnification ×200.

The pathological examination revealed a 35 mm × 25 mm × 15 mm mass that was primarily gray-white. The cut surface presented a fusion of multiple nodules, and necrosis. Under low-power magnification, the lymph node showed effacement of the nodal architecture by an infiltration of mostly large epithelioid neoplastic cells. They were arranged in patches and nests pattern (**Figure 1A**). The neoplastic cells were brightly eosinophilic cytoplasm, prominent oval nuclei with coarse chromatin, and distinct nucleoli. Pathological mitotic figures were easily found (10 mitoses per 10 high-power field) (**Figure 1B**). Partial areas showed patchy necrosis (**Figure 1C**), and multiple intravascular tumor thrombi can be seen in the lymph node capsule and surrounding adipose tissue (**Figure 1D**). Immunohistochemistry, the tumor cells were positive for CD21 (**Figure 2A**), CD23 (**Figure 2B**), CD30 (**Figure 2C**), CD68, and Vimentin. Staining for cytokeratin, P63, EMA, CD20, Pax-5, CD79a, CD3, CD5, CD4, Granzyme B, CD138, CD38, MUM-1, Kappa, Lamda, ALK, D2-40, and S-100 were negative. The Ki-67 proliferation index was approximately 50%.The abovementioned data were summarized in **Table 1**. Chromogenic *in situ* hybridization showed strong and uniform nuclear positivity of EBER in the neoplastic cells (**Figure 2D**). Based on pathological findings, the diagnosis of EBV+ epithelioid FDCS (high grade)was approved.

**Table 1:** Immunohistochemical results and technical data in text.

Antibody	Result	Manufacturer	Species	Clone
ALK	negative	MXB	mouse	MX064
CD3	negative	MXB	mouse	MX036
CD4	negative	MXB	rabbit	SP35
CD5	negative	MXB	mouse	MX052
CD20	negative	MXB	mouse	L26
CD21	diffuse positive	MXB	mouse	MX019
CD23	patchy positive	MXB	rabbit	SP23
CD30	diffuse positive	MXB	mouse	MX080
CD38	negative	MXB	mouse	MX044
CD68	patchy positive	MXB	mouse	MXO75
CD79a	negative	MXB	mouse	MX076
CD138	negative	MXB	mouse	Ml15
cytokeratin	negative	MXB	mouse	MX005
D2-40	negative	MXB	mouse	D2-40
EMA	negative	MXB	mouse	E29
Granzyme B	negative	MXB	mouse	GZB01
Kappa	negative	MXB	rabbit	**/**
Lamda	negative	MXB	rabbit	**/**
MUM1	negative	MXB	mouse	MX093
Pax5	negative	MXB	mouse	MX017
P63	negative	MXB	mouse	MX013
S-100	negative	MXB	mouse	4C4.9
Vimentin	positive	MXB	mouse	MX034
Ki67	50% positive	MXB	rabbit	MXR002

ALK, anaplastic lymphoma kinase; EMA, epithelial membrane antigen; MUM-1, multiple myeloma oncogene 1; MXB, Maixin Biotech Company (Fuzhou, China).

## Discussion

FDCS is an uncommon malignancy of follicular dendritic cells, which is derived from mesenchymal stem cells, accounting for <1% of all tumors (4). Among the reported FDCS, one-third occurs in lymph nodes. Nodal disease most often affects the cervical nodes. It is generally believed that most tumors are low-grade sarcomas. A few numbers of tumors are classified as high-grade tumors due to obvious tumor cell atypia, high mitotic counts, necrosis, metastasis, and recurrence. Immunohistochemistry is essential in confirming the diagnosis. They variably express two or more follicular dendritic markers, namely, CD21, CD23, CD35, D2-40, clusterin, and CXCL13, but at the same time, they can also abnormally express other immunohistochemical markers, such as EMA, keratin, CD4, CD30, S-100, SMA, and CD20 (5, 6). Owing to its rarity and wide pathological characteristic, it often makes the diagnosis of FDCS more challenging.

Herein, we reported a case of EBV-positive epithelioid FDCS that occurred in a cervical lymph node with high-grade morphological features, and abnormal expression of CD30, which posed a huge challenge to our differential diagnosis. Epithelioid tumor cells appear in lymph nodes with numerous intravascular thrombi and diffuse expression of EBER. Differential diagnosis of an undifferentiated carcinoma was uppermost. However, in tumor cells, epithelial markers, such as keratin, P63, and EMA, were absent. In addition to the cervical lymph nodes, the other lymph nodes of the patient’s body were also enlarged. Therefore, lymphoma was also considered in our initial diagnosis. Moreover, the patient was diagnosed with plasmablastic lymphoma (PBL) in the external hospital before. PBL is more common in patients with immune deficiency or immunosuppression but also in elderly presumptive immunosenescence (7). It shows a morphological spectrum varying from diffuse and cohesive proliferation of cells; many of them are large, with round nuclei and prominent nucleoli. Furthermore, EBER is positive in 60%–75% of PBL, and CD30 is frequently expressed. The above clinical pathological features seemed to explain some of the morphology in the case. PBL is characterized by CD20 and PAX5 negativity together with the expression of CD38, CD138, and MUM1/IRF4 plasmacytic differentiation markers. In our case, although tumor cells did not express any B-cell marker, there was no evidence to suggest their differentiation into plasmacytic. While PBL can show de-expression of all plasma cell markers, this situation is very rare (8). In addition, PBL is a quite invasive tumor, with the Ki-67 proliferation index usually very high (> 90%). However, in this case, the Ki-67 proliferation index was only 50%. Another type of lymphoma cannot be excluded—anaplastic large cell lymphoma (ALCL). ALCL is a rare subtype of peripheral/mature T-cell lymphomas, with diffuse and strong expression of CD30. Aberrant loss of pan-T-cell antigens is typical. The hallmark cells are usually numerous and large, which can mimic metastatic tumors (9). In our case, CD3 and CD5 were negative, but CD30 was diffusely expressed. ALCL is consistently negative for EBV, but EBV-associated ALCL has been sporadically reported, and even ALK-negative is more common in older adults (10). So far, the diagnosis of the tumor is confusing. After careful observation of the histological features, we reconsidered our differential diagnosis to include FDCS. The subsequent immunohistochemistry showed the clear differentiation direction of the tumor and strong expression of CD21 and CD23. The diagnosis of FDCS was imminent.

There are two types of FDCS: conventional FDCS and EBV+ inflammatory FDCS. The latter is always a low-grade malignant tumor, and its biological behavior is often relatively indolent. Compared with conventional type, it is more common in women and usually an isolated disease with a better prognosis. EBER positivity is an important clue for the diagnosis of the tumor (11). Our patient presented with multiple lymphadenopathy throughout the body; histology showed that the tumor cells were abundant, and there were obvious atypia, numerous mitoses, and necrosis. These were all characteristics of high-grade FDCS. The clinicopathological features were inconsistent with EBV+ inflammatory FDCS. The final histopathological diagnosis of lymph node EBV+ epithelioid FDCS was rendered. Conventional FDCS is now thought to be rarely associated with EBV (2); to determine whether EBV is involved in the molecular pathogenesis or is just a concomitant phenomenon during tumor development, more cases are needed for further study.

FDCS with clinical evolution is characterized by local recurrences in 28% and distant metastasis in 27% of cases (12). Common site for metastasis include the lymph nodes, lung, and liver. Complete surgical excision is the best standard of treatment; chemotherapy or radiotherapy should be associated. At present, the patient has not completed the planned chemotherapy cycle, and his prognosis is worrying. We will continue to follow up on changes in his condition.

## Conclusion

FDCS is a malignant tumor with strong heterogeneity in histological morphology and immunohistochemical phenotype. Therefore, it is crucial to perform a thorough diagnostic work-up to confirm an accurate diagnosis. Herein, we report a case of EBV+ epithelioid FDCS in a lymph node with abnormal expression of CD30, further expanding the histological characteristics of this tumor entity and providing valuable diagnostic clues for a comprehensive understanding of the pathological characteristics of this tumor.

## Histological assessment

All tissue specimens were fixed with 10% formalin and embedded in paraffin after routine processing. Tissue sections (3–4 µm) were stained with hematoxylin and eosin for subsequent microscopic examination.

## Immunohistochemistry analysis

The antibodies (predilute) and kits were purchased from Maixin Biotech Company (Fuzhou, China) and used according to the manufacturers’ instructions. The Roch automatic immunohistochemistry instrument (BenchMark ULTRA) was used. All immunostainings were performed as previously described, and appropriate positive and negative controls were employed (13).

## *In situ* hybridization

EBV status was evaluated using *in situ* hybridization with Epstein–Barr virus-encoded RNA (EBER) ISH kit (Roche, BenchMark ULTRA and Ventana). The developer used Red Counterstain II. We used known nasopharyngeal carcinoma specimens as positive controls (14).

## Patient perspective

Written informed consent was obtained from the participant for the publication of any potentially as identifiable images or data included in this article. This study was approved by the ethics committee of Guiqian International General Hospital.

## Data availability statement

The original contributions presented in the study are included in the article/supplementary material. Further inquiries can be directed to the corresponding author.

## Ethics statement

The manuscript presents research on animals that do not require ethical approval for their study. Written informed consent was obtained from the individual(s) for the publication of any potentially identifiable images or data included in this article.

## Author contributions

YC: Writing – original draft. XH: Conceptualization, Writing – original draft. XZ: Investigation, Writing – original draft. YX: Methodology, Writing – original draft. DG: Writing – review & editing.
